# HyPER: Region-specific hypersampling of fMRI to resolve low-frequency, respiratory, and cardiac pulsations, revealing age-related differences

**DOI:** 10.1016/j.neuroimage.2025.121502

**Published:** 2025-10-01

**Authors:** Adam M. Wright, Tianyin Xu, Yunjie Tong, Qiuting Wen

**Affiliations:** a Department of Radiology and Imaging Sciences, Indiana University School of Medicine, Indianapolis, IN, USA; b Weldon School of Biomedical Engineering Department, Purdue University, West Lafayette, IN, USA

**Keywords:** Hypersampled fMRI, Cardiac pulsation, Low-frequency oscillation, Respiratory oscillation, Brain physiological oscillations, Aging

## Abstract

Resting-state functional MRI (fMRI) signals capture physiological processes, including systemic low-frequency oscillations (LFOs), respiration, and cardiac pulsations. These physiological oscillations—often treated as noise in functional connectivity analysis—reflect fundamental aspects of brain physiology and have recently been recognized as key drivers of brain waste clearance. However, these critical physiological signals are obscured in fMRI data due to slow sampling rates (typical repetition time (TR) > 0.8 s), which cause cardiac signal to alias into lower frequencies. To resolve physiological signals in fMRI datasets, we leveraged fast cross-slice sampling within each TR to hypersample the fMRI signal. A key novelty of this study is the development of a region-specific hypersampling approach, called HyPER (Hypersampling for Physiological signal Extraction in a Region-specific manner). HyPER enhances temporal resolution within coherently pulsating vascular and tissue compartments, including the major cerebral arteries, the superior sagittal sinus (SSS), gray matter (GM), and white matter (WM). This study is structured in three parts: (1) We developed and validated the HyPER approach using fast fMRI from a local dataset in four regions of interest: the major cerebral arteries, SSS, GM, and WM. (2) We applied this approach to the publicly available Human Connectome Project-Aging (HCP-A) dataset (ages 36–90 years), increasing the resolvable frequency by ninefold—from 0.625 Hz to 5.625 Hz—enabling clear separation of cardiac, respiration, and LFO oscillations. (3) We investigated how brain physiological pulsations change with age. Our findings revealed an age-related increase in cardiac and respiratory pulsations across all brain regions, likely reflecting an increased vessel stiffness and reduced dampening of high-frequency pulsations along the vascular network. In contrast, LFO pulsations generally declined with age, suggesting reduced vasomotion in the older brain. In summary, we demonstrated the feasibility and reliability of a region-specific hypersampling technique to resolve physiological pulsations in fMRI. This method can be broadly applied to existing fMRI datasets to uncover hidden physiological pulsations and advance our understanding of brain physiology and disease-related alterations.

## 1. Introduction

Cerebral physiological dynamics—such as systemic low-frequency oscillations (LFOs), respiration, and cardiac pulsations—account for a significant portion of resting-state functional MRI (fMRI) signal variance (Birn et al., 2008; Chang et al., 2009; Shmueli et al., 2007). Often treated as noise in functional connectivity studies (Liu, 2016; Tsvetanov et al., 2015), these signals offer valuable insights into cerebral physiology (Raitamaa et al., 2021; Tong et al., 2017, 2014). Recent discoveries of brain waste clearance mechanisms highlight the importance of brain pulsations, identifying them as key drivers of cerebrospinal fluid (CSF) circulation and waste removal (Fultz et al., 2019; Holstein-Rønsbo et al., 2023; Mestre et al., 2018; Wright et al., 2024a). Ultrafast fMRI, with a repetition time (TR) of 100 ms, has enabled the study of these crucial physiological phenomena and waste clearance in aging, sleep, and disease (Helakari et al., 2022; Kiviniemi et al., 2016; Raitamaa et al., 2021; Rajna et al., 2021). However, such high sampling rates require specialized 3D sequences with sparse k-space sampling, which are not widely available. Even with multiband (MB) acceleration, conventional 2D fMRI TRs typically exceed 800 ms, leading to spectral aliasing, where higher-frequency signals (i.e., cardiac pulsations) fold into lower-frequency bands (Huotari et al., 2019; Kiviniemi et al., 2005). In many scenarios, the cardiac signal will alias into the LFO and respiratory passbands, which is especially problematic in large vessels where cardiac pulsations dominate the signal fluctuations. This aliasing can drastically obscure the lower frequency bands. Developing methods to extract the hidden cardiac signal from existing fMRI datasets will not only enable the direct study of cerebral cardiac signals but also ensure LFOs and respiratory signals can be interpreted without aliasing contamination. Unlocking these physiological signals can provide a wealth of information, advancing our understanding of brain physiology.

The 2D acquisition scheme in fMRI, combined with the global nature of physiological pulsations, makes it possible to recover physiological oscillations retrospectively through hypersampling. While the TR limits the per-slice sampling rate in fMRI, the cross-slice sampling rate—TR divided by the number of slices—is much faster. This cross-slice speed can be leveraged to hypersample specific tissue or fluid compartments with synchronized pulsations. Frank et al. first explored this concept and demonstrated the feasibility of resolving respiration and cardiac pulsations in fMRI by stitching cross-slice k-space phase data (Frank et al., 2001). However, a key limitation of the k-space approach is its assumption that physiological pulsations are globally uniform across space, without accounting for spatially distinct fluid compartments that may exhibit distinct pulsation patterns, such as arteries versus brain tissue. More recently, Aslan et al. extended this concept to the image domain, refining voxel selection to vascular-rich regions to reconstruct cardiac pulsations more accurately (Aslan et al., 2019). By focusing on physiologically coherent vascular regions, they achieved significantly improved cardiac waveforms compared to using the brain-wide data.

Therefore, the key to robust fMRI hypersampling lies in grouping brain voxels into compartments that exhibit coherent physiological pulsations. Physiological pulsation patterns in the brain vary spatially: Cardiac pulsations are prominent in cerebral arteries, both cardiac and respiratory pulsations are more prominent in venous sinuses, and LFOs are most prominent in brain tissue (Raitamaa et al., 2021; Tong and Frederick, 2014). In the vascular system, cardiac and respiratory pulsations drive cerebral blood delivery and circulation (Raitamaa et al., 2021; Tong et al., 2014). In gray and white matter (GM, WM), high-resistance arterioles dampen cardiac pulsations, making vasomotion-driven LFOs the dominant physiological process (Tong et al., 2017). These spatial variations underscore the need for region-specific hypersampling to reconstruct physiological pulsations across different vascular and tissue compartments. Studying the large cerebral arteries and the SSS offers a valuable snapshot into cerebral fluid regulation. Within these vessels, LFOs, respiration, and cardiac pulsations contribute to blood circulation (Raitamaa et al., 2021; Tong et al., 2019; Vijayakrishnan Nair et al., 2022). Importantly, the blood flow regulation at the large cerebral arteries and SSS significantly contributes to intracranial pressure dynamics (Wagshul et al., 2011). Characterizing the physiological oscillations of these vessels can provide insights into both cerebral circulatory health and intracranial pressure regulation in healthy aging and disease.

Recently, we developed a robust data-driven method to segment large cerebral arteries and venous sinuses in fMRI by leveraging their unique pulsation patterns (Wright et al., 2024b). Building on this advancement, we developed a regional hypersampling approach, named HyPER (Hypersampling for Physiological signal Extraction in a Region-specific manner), to resolve three physiological pulsations—LFOs, respiratory, and cardiac—across four regions of interest: large cerebral arteries, SSS, GM, and WM. Our objectives were to: 1) Develop and validate the HyPER method; 2) Demonstrate its feasibility and generalizability using fMRI data from the publicly available Human Connectome Project-Aging (HCP-A) cohort (ages 36–90 years); 3) Investigate the impact of aging on these physiological components across the cerebral arteries, SSS, GM, and WM.

## 2. Methods

### 2.1. Human participants

This study’s data came from two cohorts: a local cohort to validate the hypersampling technique and the Human Connectome Project-Aging (HCP-A) 2.0 release to characterize brain region-specific physiological changes associated with age. The local cohort used a fast fMRI acquisition where all physiological signals were critically sampled. This allowed us to compare before and after hypersampling to ensure the processing did not alter physiological measures. All local cohort participants provided written informed consent according to procedures approved by the Institutional Committee for the Protection of Human Participants at Indiana University and Purdue University. All HCP-A participants provided informed consent (Bookheimer et al., 2019), and the institutional review board at Purdue University approved this retrospective analysis of HCP-A data.

### 2.2. Image acquisition

The local cohort was imaged with a 3T Prisma Siemens scanner and a 64-channel head-neck coil. The T1-weighted (T1-w) scan employed a 3D magnetization rapid gradient echo (MPRAGE) sequence with a voxel size of 1.0 × 1.0 × 1.0 mm^3^. The local cohort had three to four fMRI repeats per participant. The fMRI acquisition parameters were as follows: repetition time (TR) = 366 ms, echo time (TE) = 29.80 ms, flip angle (FA) = 35°, voxel size = 2.5 × 2.5 × 2.5 mm^3^, slices = 40, slice distance factor = 0 %, volumes = 500, MB factor = 8, anterior-posterior phase encoding, and an acquisition time of 3 min and 3 s. Simultaneous finger photoplethysmography (PPG) was recorded during all fMRI scans.

The HCP-A cohort was imaged with a 3T Prisma Siemens scanner and a 32-channel head-neck coil. The T1-w anatomical scan had a voxel size of 0.8 × 0.8 × 0.8 mm^3^. The fMRI acquisition parameters were: TR = 800 ms, TE = 37.0 ms, FA = 52°, voxel size = 2.0 × 2.0 × 2.0 mm^3^, slices = 72, slice distance factor = 0 %, volumes = 488, MB factor = 8, and an acquisition time of 6 min and 30 s. Repeated fMRI scans in the HCP-A cohort were conducted over two imaging sessions on separate days. For each session, a pair of scans with opposite phase encoding directions (anterior-posterior and posterior-anterior) were acquired. Simultaneous finger PPG was recorded during all fMRI scans.

### 2.3. Image processing

#### 2.3.1. Brain region of interest segmentation

This study’s regions of interests (ROIs) included the large cerebral arteries, SSS, WM, and GM. An automatic, data-driven algorithm was used to segment the large cerebral arteries, including the anterior, middle, and posterior cerebral arteries, and SSS in fMRI space (Wright et al., 2024b). The algorithm identified vascular regions by detecting voxels with reproducible cardiac pulsatility. This was achieved by randomly splitting the fMRI timepoints into two subsets, retrospectively realigning each subset to the cardiac cycle, and computing the voxel-wise correlation between the two cardiac-aligned time series. Voxels with high correlations were identified as likely vascular voxels. To separate the large cerebral arteries and SSS segmentations, general search regions for the large arteries or the SSS region were used. The cerebral artery search region was adapted from the statistical brain atlas (Dunås et al., 2017), and modified to include the anterior, middle, and posterior cerebral arteries. The SSS search region was created by manually segmenting the SSS in MNI space and dilating it in the subject’s fMRI space. This algorithm was able to locate large vascular voxels with high test-retest reproducibility (see (Wright et al., 2024b) for more details). GM and WM regions were segmented on T1-w MR images using Freesurfer (recon-all) and then transformed to fMRI space (FSL: FLIRT, nearest-neighbor interpolation).

#### 2.3.2. Hypersampling theory and implementation

The hypersampling technique achieved a higher temporal resolution by leveraging fast cross-slice sampling in 2D fMRI. In a 2D fMRI, slices are acquired sequentially or interleaved within a TR, each with a unique slice timing. With multiband excitations, the number of unique slice timings per TR is given by *N_Time_* = *N_Slice_*/*MB* where N_Slice_ is the total number of slices and MB is the multiband factor. For example, in the HCP-A fMRI, with N_Slice_ = 72 slices and MB = 8, there are N_Time_ = 72/8 = 9 unique slice timings per TR. By aligning these 9 slice timings from a coherently pulsating tissue compartment in time, we generated a hypersampled signal, improving temporal resolution from the original TR to *TR*/*N_Time_* (s). Thus, the improvement in hypersampling rate is determined *N_Time_*. In the HCP-A example, the temporal resolution improved by *N_Time_* = 9 fold, increasing the Nyquist frequency from 1/(2·*TR*) = 0.625 *Hz* to 1/(2·*TR/N_Time_*) = 5.625 *Hz*.

**Fig. 1.**
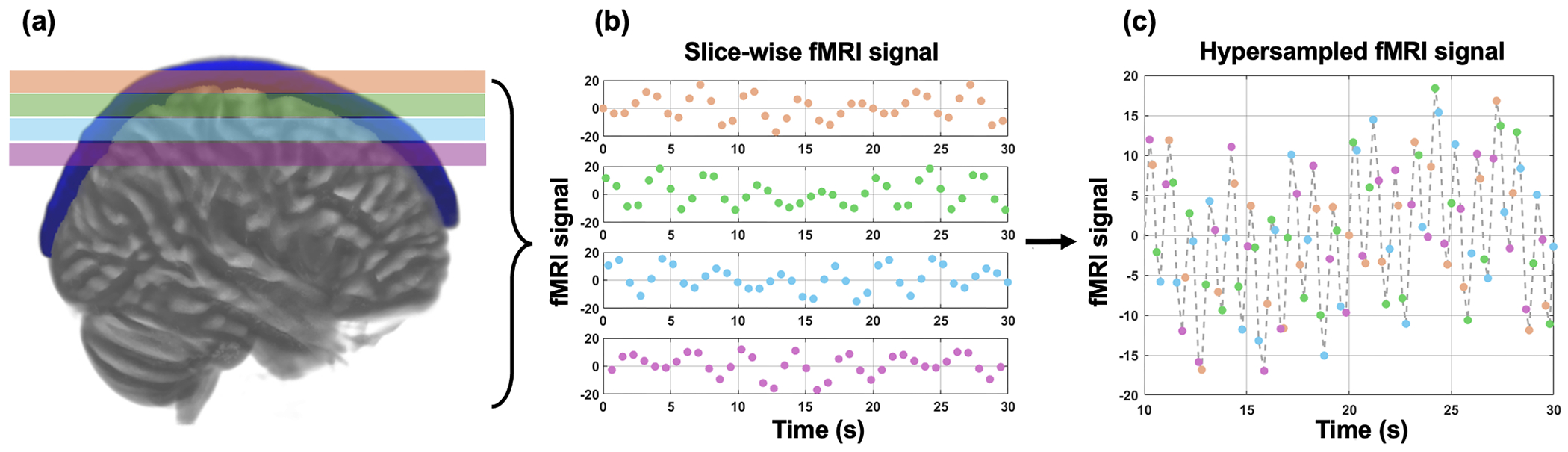
Schematic overview of the hypersampling method with *N_Time_* = 4. **(a)** Hypersampling was performed within a specific region exhibiting coherent physiological pulsations, which is shown here as the superior sagittal sinus (SSS, blue). **(b)** Slice-wise fMRI signals from the SSS were averaged, demeaned, and time-shifted according to each slice’s acquisition time. (**c**) These time-shifted SSS signals were then combined to create a hypersampled signal, effectively increasing the sampling rate by 4-fold. **Note:** This illustration is based on simulated data and does not use actual data.

The region-specific hypersampling approach was completed in three steps, as illustrated in Fig. 1, using the SSS as an example:

1. Segmentation of ROIs: ROIs of the same tissue type were segmented (see Section 2.3.1), and hypersampling was applied to each ROI individually (Fig. 1a).
2. Generation of*N_Time_* temporal signals from unique slice timings: Voxel-wise time series were detrended (matlab: detrend.m, first-order) to remove signal variations from non-physiological sources, such as B0 field inhomogeneity and scanner drift effects. Voxels sharing the same slice timings were averaged, producing one time series per unique slice timing, totaling *N_Time_* temporal signals (Fig. 1b).
3. Reconstruction of hypersampled signal: The *N_Time_* time series were ordered according to their slice timings. These time-shifted temporal signals were then aligned to generate one hypersampled signal for each ROI (Fig. 1c).

#### 2.3.3. Physiological bandpower calculation

The strength of physiological oscillations were quantified with the bandpower within physiological frequency bands—LFOs (0.01–0.1 Hz), respiratory (0.2–0.4 Hz), and cardiac (heartbeat frequency±0.15 Hz). Bandpower was computed by estimating the hypersampled signal’s power spectral density (PSD, matlab: periodogram.m), and integrating the PSD over the relevant frequency range (matlab: bandpower.m). The hypersampled bandpower was referred to as the, “Power of Hypersampled ROI”.

### 2.4. Hypersampling comparison with fast fMRI

We used the locally acquired fast fMRI to test and determine the impact of hypersampling on physiological power, as its acquisition was fast enough to resolve cardiac frequencies. The hypersampling pipeline (Section 2.3.2) was applied to the local fMRI dataset with TR = 0.366 s, *N_Slice_* = 40, MB = 8, *N_Time_* = *N_Slice_*/*MB* = 5, resulting in an original sampling rate of 1/*TR* = 2.73 Hz (Nyquist frequency = 1.365 Hz) and a hypersampled sampling rate of 1/(*TR* /*N_Time_*) = 13.66 Hz (Nyquist frequency = 6.83 Hz). The mean timeseries of each ROI was used to calculate each physiological bandpower (as calculated in 2.3.3) and compared with the hypersampled bandpower. The fast fMRI data bandpower was referred to as the “Power of ROI-mean Timeseries”.

### 2.5. Impact of pulse propagation on hypersampling

Physiological pulses propagate through the brain with phase delays, which deviate from the assumption of coherent pulsation, potentially affecting hypersampling results. To assess the impact of physiological pulse propagation on the hypersampled bandpower estimates, we compared them with voxel-wise physiological bandpower from the locally acquired fast fMRI. Since voxel-wise power is unaffected by propagation, it served as a reliable reference. The voxel-wise physiological bandpower was calculated for each voxel-wise timeseries using the same methods in 2.3.3. The mean of the voxel-wise physiological bandpower within an ROI was calculated, referred to as the “ROI-mean of Voxel-wise Power”, and compared with the hypersampled bandpower. We hypothesize that if pulse propagation had no impact on hypersampling, the ROI-mean voxel-wise bandpower should closely correlate with the hypersampled bandpower; Conversely, if propagation introduced phase delays across voxels, the hypersampled results would exhibit reduced power due to signal cancellation.

### 2.6. fMRI quality control criteria

Each fMRI dataset underwent quality control for scan motion and vessel segmentation. Because hypersampling relies on exact slice timing, it is incompatible with motion correction and requires the exclusion of scans with excessive motion. Motion estimates were calculated using FSL’s mcflirt, and scans with more than 15 % of fMRI volumes showing absolute motion exceeding 1 mm were excluded. Additionally, as vessel segmentation relies on PPG signal, datasets with poor PPG quality and unreliable vessel segmentation were excluded. PPG quality was automatically evaluated using an in-house function that assessed the proportion of total signal power within the cardiac frequency band, following the methodology described in (Wright et al., 2024b). Lastly, scans were excluded if an ROI spanned fewer than half of the unique slice timings (i.e., an artery or SSS ROI spanned four or fewer slice timings in HCP-A cohort, where N_Time_ = 9).

### 2.7. Statistical analysis

Linear mixed-effects models were used to assess the effects of age and biological sex on physiological pulsation bandpower across various brain regions in HCP-A. Due to the non-linear relationship between bandpower and age, a log transformation was applied to improve linearity. The models accounted for random intercepts by participant to control for within-subject variability from repeated scans. The linear mixed-effects model was defined as follows:

$$y_{ij}=\beta_{0}+\beta_{1}age_{j}+\beta_{2}\text{biological}\;{\text{sex}}_{j}+u_{i}+\in_{ij}$$

where *y_ij_* represents the log-transformed bandpower (specific to ROIs and physiologic components) for each participant *i* and repeated scan *j*, *u_i_* is the random effect accounting for between-subject variability, and *ϵ_ij_* is the residual error accounting for within-subject variability. The models were implemented using the lme4 and lmerTest packages in R. Multiple comparisons across the three frequency bands within each ROI were corrected with false discovery rate (FDR), with statistical significance set at *p* < 0.05.

## 3. Results

### 3.1. Participants and demographics

All local dataset scans passed the quality checks, resulting in 19 scans from 5 participants (26.8 ± 5.4 years, 40 % Men). Within the HCP-A dataset, 218 out of 2856 fMRI scans were excluded due to excessive motion, 672 scans were excluded due to poor PPG quality leading to unreliable vessel segmentation, and 43 were excluded because their vessel ROIs spanned fewer than half of the unique slice timings. Ultimately, 1923 scans from 609 HCP-A participants (59.1 ± 14.7 years, 43 % Men) were included in this analysis.

### 3.2. Hypersampled fMRI resolved the cardiac frequency in HCP-Aging

**Fig. 2.**
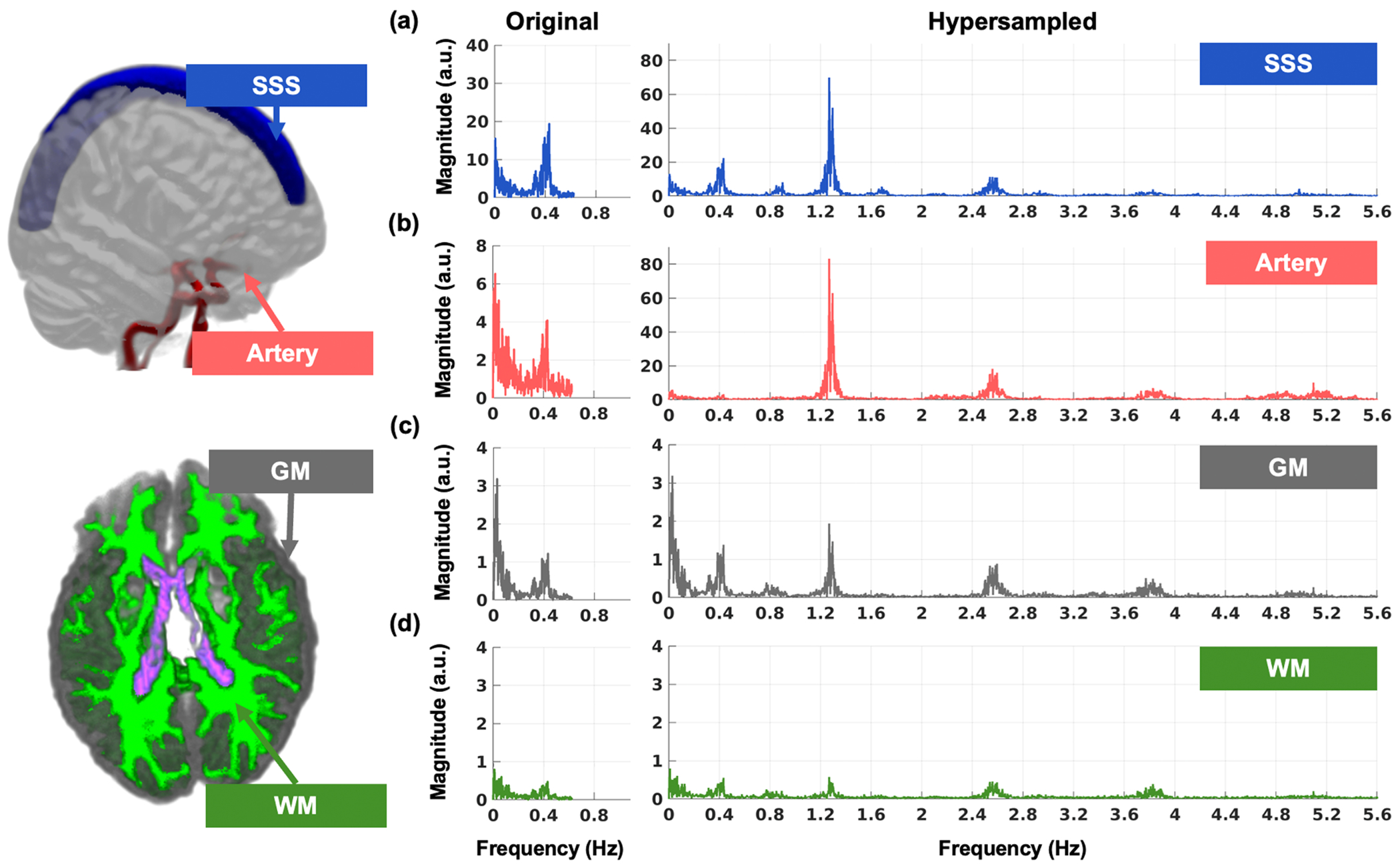
Hypersampling successfully resolved the cardiac frequency in a representative HCP-A dataset across four regions of interests (ROIs). Hypersampling increased the sampling rate by 9-fold $\left(\frac{N_{Slice}}{MB}=\frac{72}{8}=9\right)$ raising the Nyquist frequency from 0.625 Hz to 5.625 Hz. The frequency spectra before (left) and after hypersampling (right) demonstrate that the previously aliased cardiac frequencies were successfully resolved across all ROIs: **(a)** superior sagittal sinus (SSS), **(b)** large cerebral arteries, **(c)** gray matter (GM), and **(d)** white matter (WM).

In the HCP-A cohort, hypersampling enhanced the fMRI signal’s sampling rate by a factor of 9 $\left(\frac{N_{Slice}}{MB}=\frac{72}{8}\right)$, increasing the Nyquist frequency from 0.625 Hz to 5.625 Hz. This enabled the successful resolution of cardiac pulsations, as illustrated in a representative participant in Fig. 2. After hypersampling, the previously unresolvable cardiac component became evident in all ROIs. The cardiac frequency emerged as the dominant frequency component in the large cerebral arteries and the SSS, reflecting strong cardiac-driven pulsations. LFOs remained dominant in the GM and WM. However, a discernible cardiac frequency component was revealed after hypersampling.

### 3.3. Strong agreement in physiological power between hypersampled and fast fMRI

In the local cohort, hypersampling increased the sampling rate 5-fold $\left(\frac{N_{Slice}}{MB}=\frac{40}{8}\right)$, elevating the Nyquist frequency from 1.37 Hz to 6.84 Hz. Since the original sampling already resolved the cardiac frequency, it enabled a direct comparison of physiological pulsation power before and after hypersampling.

**Fig. 3.**
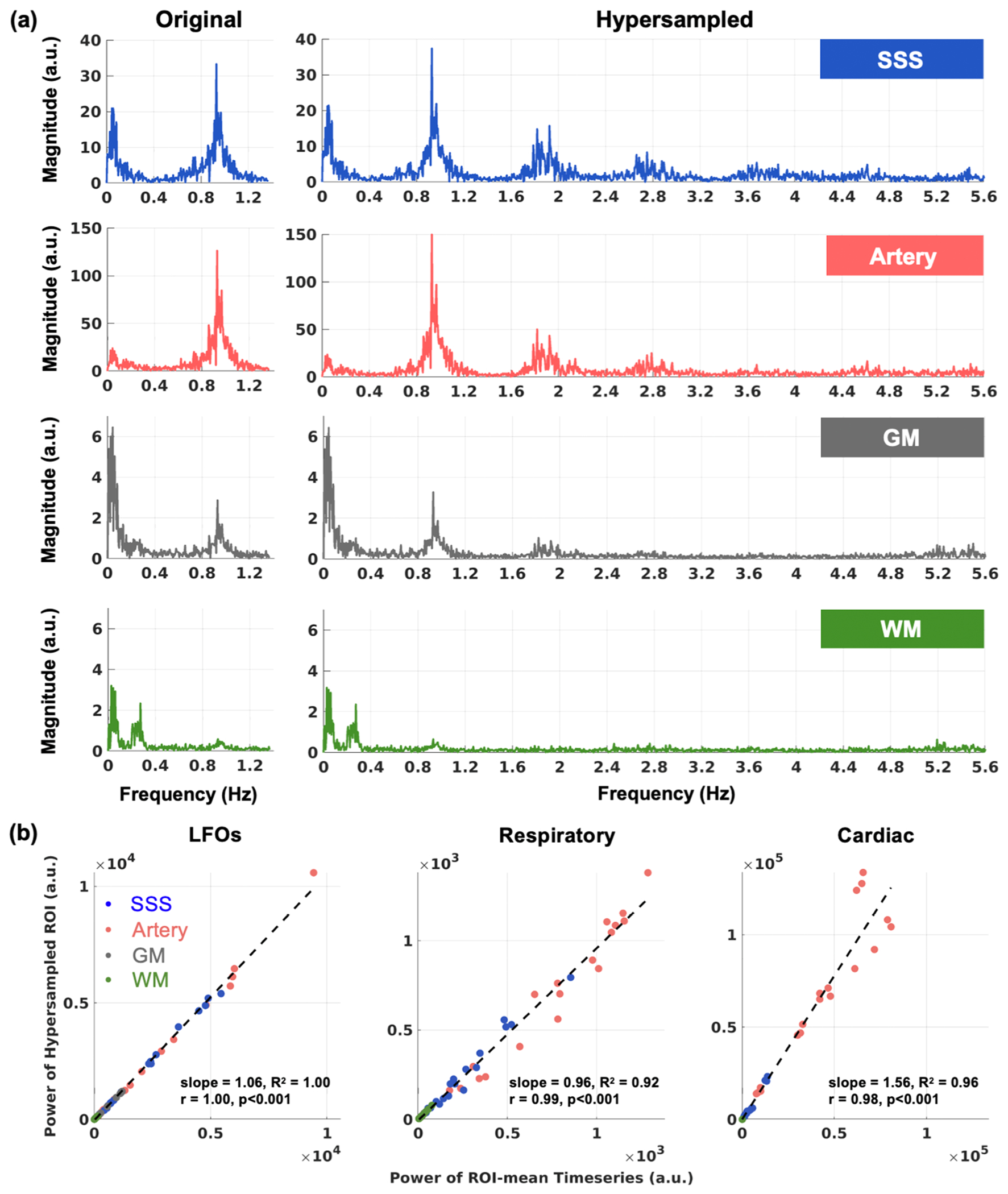
The hypersampling method was validated with fast fMRI datasets (TR=366 ms). The fast fMRI resolved three physiological pulsations without hypersampling, enabling a direct comparison of physiological power before and after hypersampling. **(a)** Power spectra from a representative participant show agreement between the original fMRI signal (Nyquist frequency = 1.37 Hz, left) and the hypersampled fMRI signal (Nyquist frequency = 6.84 Hz, right) across brain regions. **(b)** A strong agreement in absolute power was observed between original and hypersampled data across all physiological frequency bands (19 total fMRI scans from 5 participants). **Abbreviations:** LFOs – low frequency oscillations, SSS – superior sagittal sinus, GM – gray matter, WM – white matter.

Across all ROIs, the frequency spectrum in the hypersampled fMRI closely matched that of the original fast fMRI (Fig. 3a). The physiological power measures from the hypersampled fMRI data were strongly correlated with the power of the ROI-mean timeseries from the fast fMRI (Fig. 3b, *r* > 0.95, *p* < 0.001). The linear relationship varied by physiological pulsation. Specifically, the LFO and respiratory power had regression slopes close to 1 (slope=1.06 and 0.96, respectively), while the cardiac power was approximately 1.5 times higher (slope=1.56) in the hypersampled fMRI. The ROI-specific regression results showed similar trends and were summarized in Supplemental Figure 1. The higher cardiac power observed in hypersampling compared with the original fast fMRI time series arises from its ability to better recover the signal oscillations by accounting for slice timing. In contrast, the ROI-mean operation ignores slice timing differences, leading to temporal averaging across slices. This averaging causes greater destructive interference for rapidly varying signals, such as cardiac pulsation, than for lower frequency signals such as LFOs and respiratory oscillations. For example, in fMRI acquisitions, neighboring slices capture different phases of the cardiac cycle, so averaging across slices combines multiple cardiac phases that destructively interfere. Consequently, cardiac power is underestimated in the ROI-mean signal. This effect is further illustrated in the supplemental analysis (see Supplemental Material 3.3.2, Supplemental Figure 2).

### 3.4. Impact of pulse propagation on hypersampling power estimates

**Fig. 4.**
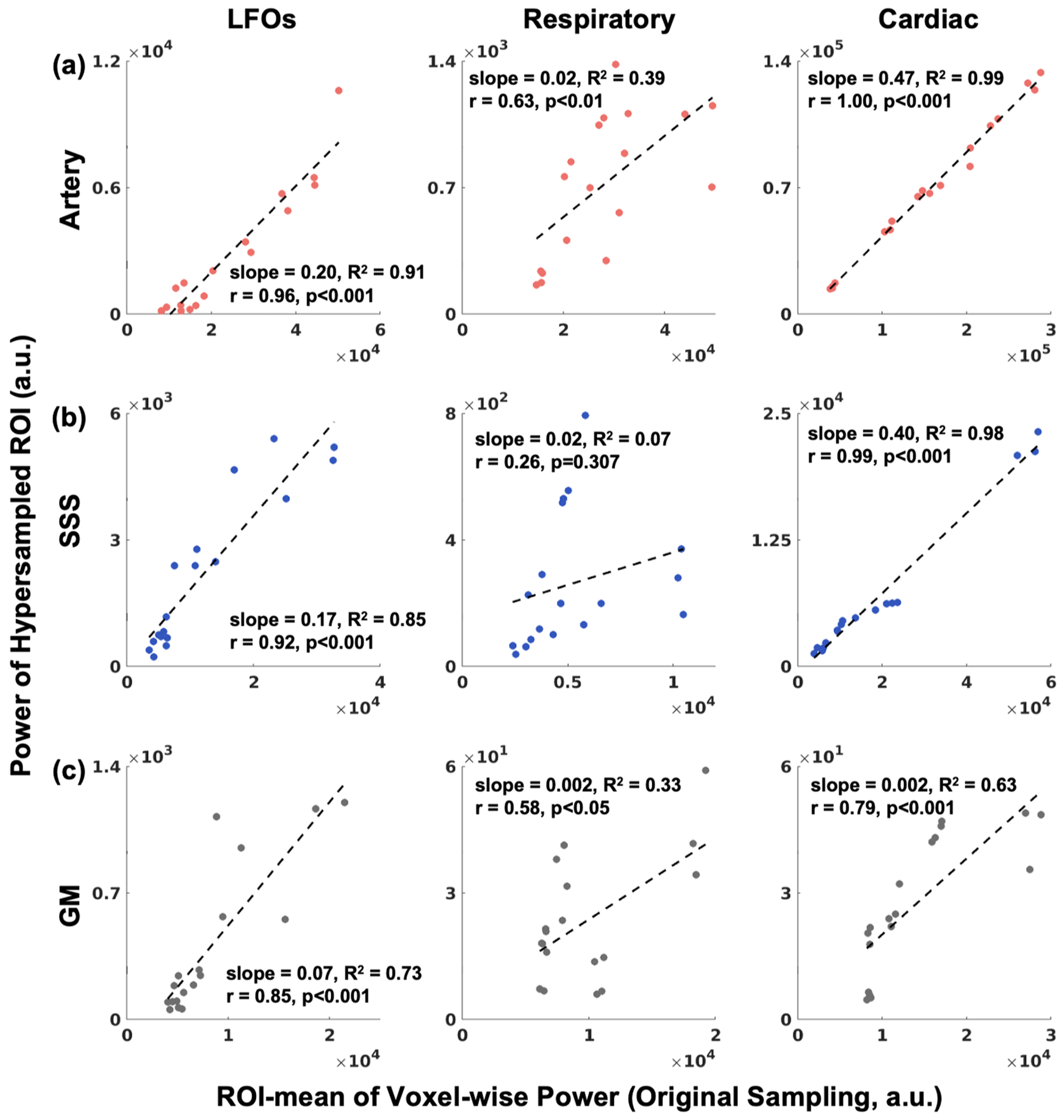
Impact of physiological pulse propagation assessed by comparing hypersampled ROI bandpower (Hypersampled ROI power) with voxel-wise physiological bandpower from the locally acquired fast fMRI (ROI-mean Voxel-wise Power). The physiological oscillations are shown left to right (LFOs, respiration, cardiac), and ROIs are shown top to bottom: (a) Artery, (b) SSS, (c) GM. The linear relationship between hypersampled ROI power and the mean voxel-wise power was generally strong for LFOs and cardiac power (0.79 ≤ *r* ≤ 1.00), but weaker for respiratory power (0.26 ≤ *r* ≤ 0.63). **Abbreviations:** LFOs – low frequency oscillations, SSS – superior sagittal sinus, GM – gray matter, WM – white matter.

The linear relationship between hypersampled ROI power and the mean voxel-wise power varied across physiological pulsations, with LFO and cardiac power showing a better linear relationship than respiration (Fig. 4). Specifically, the LFO and cardiac power from hypersampled fMRI were strongly correlated with voxel-wise fast-sampling fMRI power in the arteries, SSS, and GM (LFO: Artery, *r* = 0.89; SSS, *r* = 0.92; GM, *r* = 0.85; cardiac: Artery, *r* = 1.00; SSS, *r* = 0.99; GM, *r* = 0.79; all FDR-corrected *p* < 0.001). Respiratory power was moderately correlated in arteries (*r* = 0.63, *p* < 0.01) and GM (*r* = 0.58, *p* < 0.05), but weakly correlated in the SSS (*r* = 0.26, *p* < 0.05). In WM, physiological power was moderately correlated in the LFOs (*r* = 0.63, *p* < 0.01) and strongly correlated in respiratory (*r* = 0.94,*p* < 0.001) and cardiac power (*r* = 0.85, *p* < 0.001), see Supplemental Figure 4.

### 3.5. Age-related differences in hypersampled power spectra in HCP-Aging

**Fig. 5.**
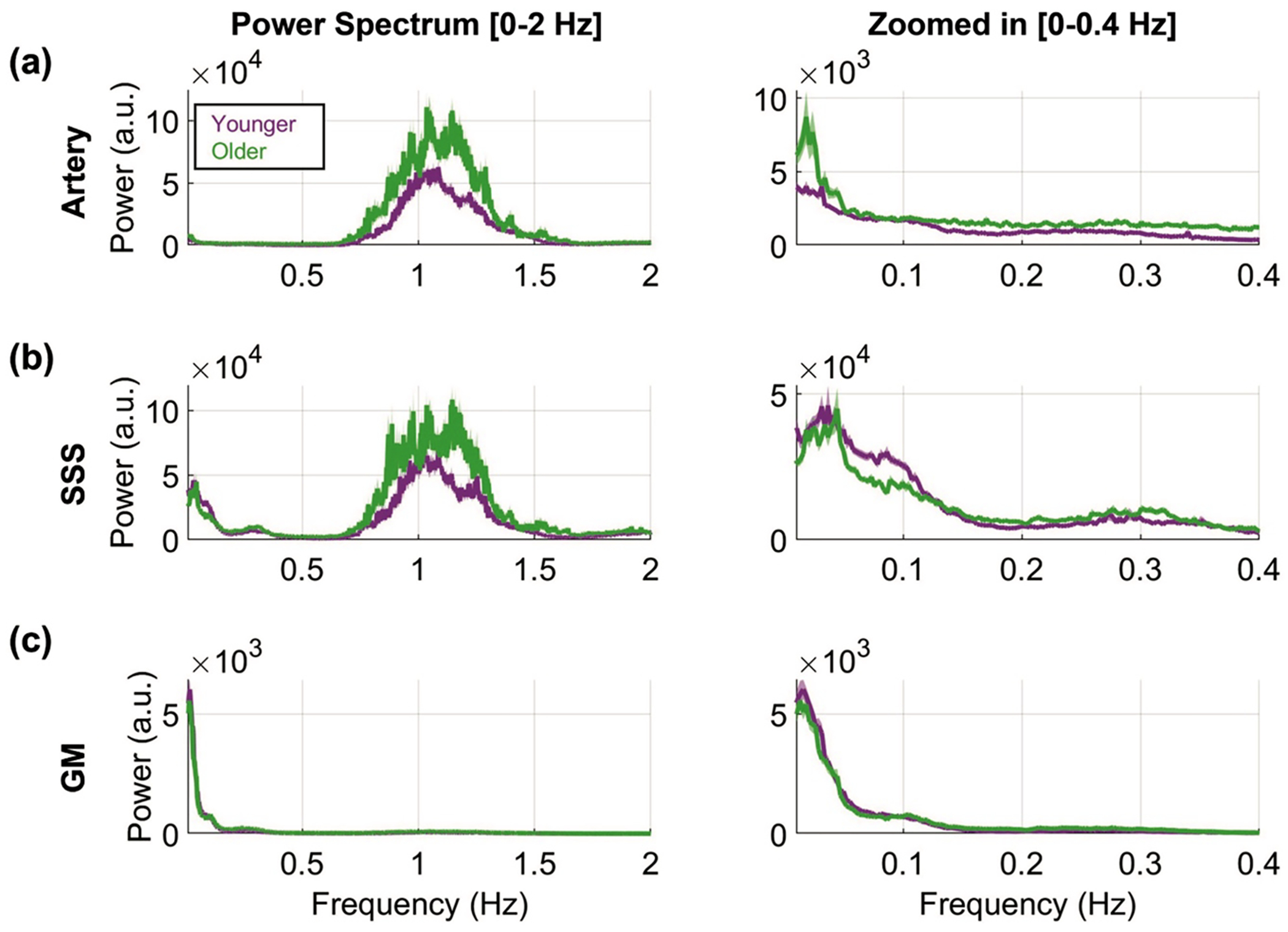
Hypersampled power spectra in HCP-A between younger (49.7 ± 8.2 years) and older (75.7 ± 6.7 years) groups across key brain regions: **(a)** large cerebral arteries, **(b)** superior sagittal sinus (SSS), and **(c)** gray matter (GM). The mean (solid line) and standard error (shaded region) are displayed. The power spectrum from 0–2 Hz (left) and zoomed in from 0–0.4 Hz (right).

When grouped into younger (age <65 years, 49.7 ± 8.2 years, *N* = 388, 41 % men) and older (age ≥ 65 years, 75.7 ± 6.7 years, *N* = 221, 46.1 % men) cohorts, hypersampled HCP-A data revealed age-related changes in physiological pulsations across all ROIs (Fig. 4). In the large cerebral arteries, both groups displayed cardiac-dominant pulsations and the older group had elevated power across all frequency bands (Fig. 5a). In the SSS, cardiac pulsations remained dominant in both groups, while LFO and respiratory power were slightly elevated compared with the arteries. Compared to the young group, the older group had increased respiratory and cardiac power but decreased LFO power (Fig. 5b). In the GM, LFOs were dominant in both age groups, though older participants exhibited decreased LFO power (Fig. 5c). WM showed a similar trend, which is summarized in Supplemental Figure 5a.

**Fig. 6.**
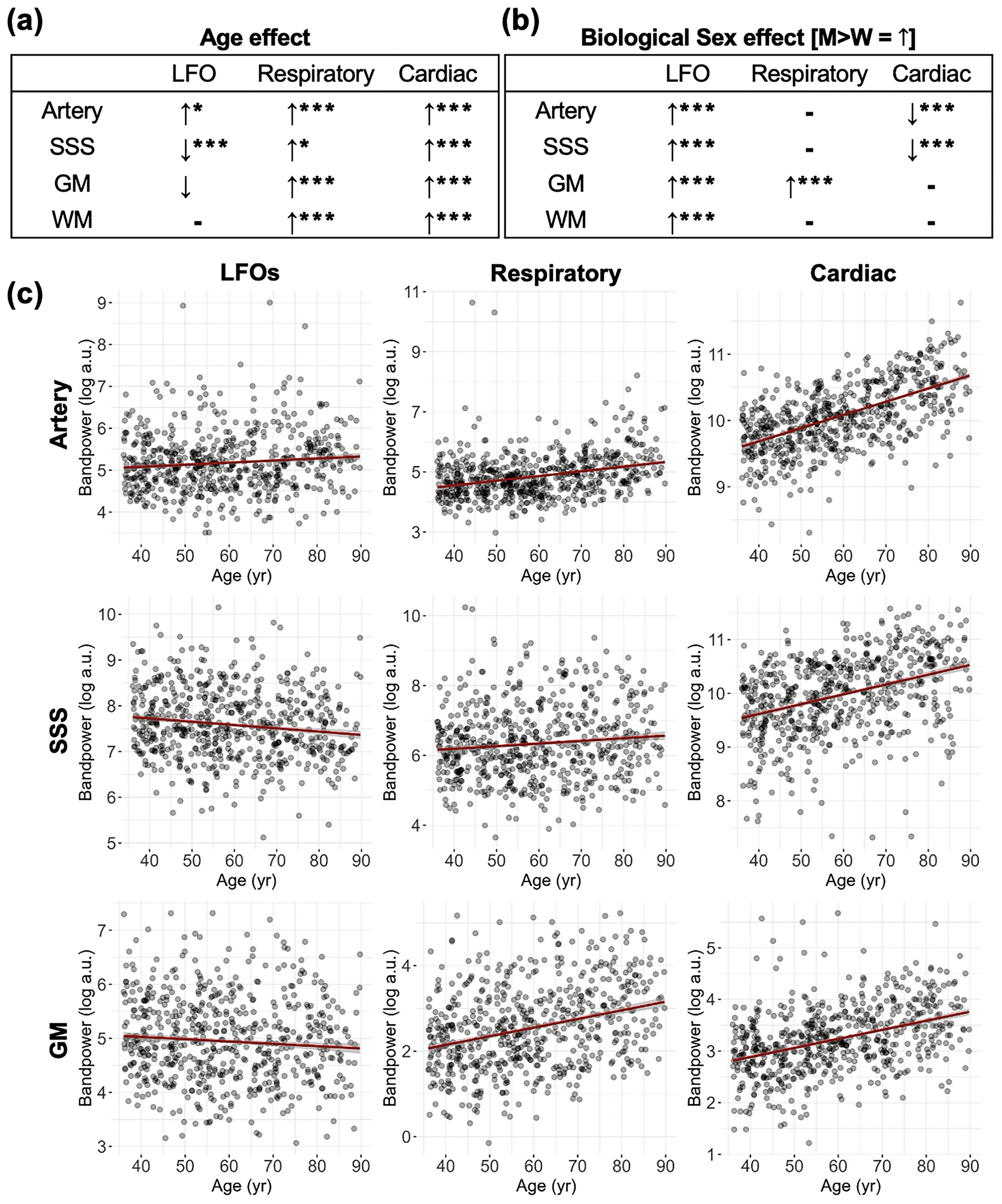
Age and biological sex effects on physiological pulsation power across key brain regions. **(a)** Summary of age effects and **(b)** biological sex effects (men compared to women) from linear mixed-effects models with FDR correction. **(c)** Scatter plots show the relationship between age and bandpower across brain regions. **Abbreviations:** LFOs – low frequency oscillations, SSS – superior sagittal sinus, GM – gray matter, WM – white matter. *p < 0.05, **p < 0.01, ***p < 0.001.

Age and biological sex effects on physiological bandpower across various brain regions were assessed using linear mixed-effects models of log-transformed bandpower (Fig. 6; statistics are summarized in Supplemental Table 1). Age was positively associated with cardiac and respiratory power in the large cerebral arteries, SSS, GM, and WM (FDR *p* < 0.05 for all comparisons; Fig. 6a). The most pronounced age effect was on cardiac power, where power increased with age in all brain regions (FDR *p* < 0.001; Fig. 6c). LFO power showed region-dependent associations with age, decreasing in SSS (FDR *p* < 0.001) and GM (trending lower, FDR *p* = 0.054) but increasing in arteries (FDR *p* < 0.05). Scatter plots for WM are summarized in Supplemental Figure 5b.

Biological sex was significantly associated with physiological bandpower (Fig. 6b, boxplots in Supplemental Figure 6). Compared to women, men exhibited lower cardiac power in the large cerebral arteries and SSS (FDR *p* < 0.001 for both), higher respiratory power in GM (FDR *p* < 0.001), and higher LFO power across all ROIs (FDR *p* < 0.001 for all comparisons).

## 4. Discussion

In this work, we developed HyPER, a regional hypersampling method that recovers hidden cardiac pulsations and prevents their aliasing into the LFO or respiratory frequency of the fMRI signal. Our results in both local and HCP-A data demonstrated the feasibility and reliability of this approach in recovering spatially distinct pulsation patterns within the brain. Additionally, our analysis of the HCP-A data revealed age-related alterations across all pulsation frequencies, highlighting the method’s potential to uncover new insights on brain physiology from existing fMRI data. These findings, along with the associated technical considerations, are discussed below.

### 4.1. Region-specific hypersampling reveals spatially distinct pulsation patterns

Region-specific hypersampling is critical to study spatially distinct brain oscillations. Our hypersampling results align well with the characteristic pulsation patterns in vascular and tissue compartments. Specifically, we found that cardiac pulsations dominated the large cerebral artery fMRI signal, while respirations and LFOs had minimal influence. In contrast, SSS signal dynamics were shaped by all three physiological rhythms: cardiac pulsations, respiration, and LFOs. These differences reflect the distinct mechanisms driving arterial and venous blood flow: cardiac pulsations actively propel arterial flow, whereas passive sinus compression governs SSS signal fluctuations, primarily modulated by intracranial pressure influenced by cardiac pulsations, respiration, and LFOs (Schaller, 2004; Wagshul et al., 2011). The large cerebral arteries and the SSS are the main passageways for cerebral blood circulation, making their function critical to maintaining cerebral health. Monitoring the prominent physiological oscillations within these vessels offers valuable insight into neurofluid regulation, which can reflect information on cerebral blood dynamics, intracranial pressure fluctuations, and potentially cerebrospinal fluid motion (Schaller, 2004; Wagshul et al., 2011). In comparison, LFOs were the primary contributors to signal dynamics in GM and WM, as high resistance arterioles dampen the cardiac pulsations before they propagate into brain tissue. These findings demonstrate hypersampling’s capability to reliably unalias and reconstruct physiological oscillations across all compartments, regardless of their frequency compositions.

Hypersampling enhances coherent dynamics while suppressing incoherent oscillations, provided that fMRI signals within an ROI exhibit a consistent pattern. This condition is best met in large arteries and veins, where global physiological signals are relatively homogenous across the structure. In contrast, GM and WM require further discussion, particularly for LFOs, which have both neuronal and physiological origins. Neuronal-driven fMRI signals in GM and WM are typically incoherent, as reflected in functional connectivity matrices with both positive and negative correlations. In such cases, hypersampling will suppress the incoherent neuronal fluctuations while preserving the coherent global oscillations of physiological origins. Consequently, GM/WM LFO hypersampling results mainly reflect systemic physiological LFOs. However, when coherent neuronal oscillations are present—such as during task fMRI or slow-wave sleep—hypersampling can preserve these signals as well. While the LFO components may depend on brain state, hypersampling reliably separates respiration and cardiac signals from LFOs, enabling a more precise analysis of LFOs free from these confounds.

### 4.2. Impact of physiological pulse propagation on hypersampling

The hypersampling method assumes that physiological pulsations within a brain region occur at roughly the same time. This assumption may not always hold, as Kiviniemi et al. have shown, physiological pulsations exhibit wave-like propagation across the brain (Kiviniemi et al., 2016). Differences in propagation phase would cause signal cancellation in the averaged timeseries. To evaluate the impact of this propagation, we compared the hypersampled ROI power with the voxel-wise power of fast fMRI, which is not affected by pulse propagation. We observed high correlations for LFOs and cardiac frequencies, but moderate correlations in respiratory frequencies. These results suggest that pulse propagation differentially impacts hypersampled physiological components. For LFOs and cardiac, the power measures maintained a consistent linear relationship with the ground truth, whereas respiration measures were affected by the pulse propagation.

These differences are likely due to the propagation wavelength of each physiological pulse, which is determined by its period and propagation speed (λ=Tv). The longer the propagation wavelength, the less the impact of phase shifts during spatial averaging. LFOs have long periods (~10–100 s), and cardiac pulsations propagate very fast (~1–5 m/s), resulting in long pulse wavelengths that are less sensitive to propagation-induced time shifts. In contrast, the respiratory pulse has a shorter period than LFOs and a slower propagation than cardiac pulses, resulting in a shorter wavelength and making the respiratory component more susceptible to destructive interference from pulse phase dispersion. Due to this finding, hypersampled respiratory signal is more impacted by pulse propagation than LFO and cardiac signals and should be interpreted with caution. It should be noted that the impact of propagation is not unique to hypersampling but applies to any spatial averaging of time signals, such as the conventional ROI-mean. Finally, the impact of physiological propagation discussed here is specific to fMRI hemodynamics. Other dynamic signals, such as those from dynamic diffusion-weighted imaging of hydrodynamics (Wen et al., 2025, 2023, 2022), may follow different propagation wavelengths, and their impact on hypersampling warrants further investigation.

### 4.3. Age effect on brain physiological oscillations in HCP-A

Our findings indicate that cardiac power increased with age across all brain regions. The cardiac power in fMRI represents the magnitude of pulsatile signal changes associated with the cardiac frequency (Raitamaa et al., 2021). In arteries, the fMRI signal fluctuations correspond to differences in blood velocity within a vessel, where the velocity gradient of laminar flow produces differential phase shifts across spins, reducing signal magnitude through a spin-phase phenomenon (Von Schulthess and Higgins, 1985). The increase in cardiac power in the large cerebral arteries likely reflects age-related arterial stiffening, which amplifies blood pulsatility (O’Rourke and Hashimoto, 2007; Zimmerman et al., 2021). Similarly, in brain tissue (GM and WM), the increased cardiac power may reflect an age-related reduction in the dampening of cardiac impulses (Zarrinkoob et al., 2016), resulting in a prominent pulsation that reaches the vascular networks within brain tissues. The SSS cardiac pulsation is a passive process involving mechanical compression of venous structures in response to arterial pulsations and intracranial pressure dynamics (Wagshul et al., 2011). Therefore, the age-related increase in SSS cardiac power is likely closely linked to the arterial system changes that occur with aging (Bateman et al., 2008).

We observed that LFO power was trending lower with age in GM, consistent with previous reports (Kumral et al., 2020; Zhong and Chen, 2022). The LFO decrease may reflect reduced vasomotion, which is regulated by arterioles. As the brain ages, the arterioles lose elasticity, causing disrupted rhythmic oscillations of vasomotion (Schroeter et al., 2004). The SSS mirrored the GM findings, with reduced LFO power with age. Since the SSS primarily drains blood from upstream GM, the age-related decline in GM LFO power likely contributes to the reduction in the SSS. Contrary to GM and SSS, LFOs in the large cerebral arteries increased with age. The opposite trend of LFO changes between arteries and GM suggests a more complicated LFO regulation mechanism than was observed with cardiac pulsation and warrants further investigation.

The findings in HCP-A underscore the sensitivity of fMRI to age-related physiological changes. Cardiorespiratory signal dynamics are relevant beyond aging and have been shown to serve as markers for conditions such as Alzheimer’s disease (Tuovinen et al., 2020), epilepsy (Kananen et al., 2018), and sleep (Helakari et al., 2022), as demonstrated in ultrafast fMRI studies (TR = 100 ms, 10 Hz). The developed hypersampling method extends the ability to study these physiological signals in existing fMRI datasets across a wide range of disease conditions. Our results using HCP-A demonstrate the generalizability of this approach for such explorations.

### 4.4. Technical considerations

This hypersampling method is based on a 2D EPI fMRI acquisition, where the number of slices, MB factor, and TR determine the hypersampled sampling rate expressed as *TR*/(*N_Slice_* /MB). Adjustments to these parameters change the sampling rate, each with its own tradeoffs. First, a faster TR will increase the effective sampling rate but will decrease steady-state transverse magnetization. Second, a MB factor > 2 is preferred to ensure ROIs span all unique slice timings (*N_Time_* = N_Slice_/*MB*). A low MB leads to a high number of slice timings, which may cause an ROI to miss certain slice timings, creating gaps in the hypersampled signal. For MB=1, hypersampling will only work for GM and WM if the regions span most of the slice timings. MB=1 is less effective for arteries and the SSS, which span only a small portion of the slice timings. Ideally, *N_Time_* should be equal to or less than the number of slices in the ROI to avoid missing values in the hypersampled signal. When this condition is met, hypersampling can be applied to fMRI scans with longer TRs = 2 to 3 s. We believe these considerations are valuable for future users to explore their own datasets.

### 4.5. Limitations

First, the hypersampling technique trades spatial resolution for temporal resolution. It requires signal averaging from ROIs that span enough slices, limiting its ability to be applied to small brain areas. Second, the technique requires exact slice-timing, which restricts the use of motion correction because any translation of the fMRI data would disrupt the signals’ exact acquisition time. Third, it is limited to an ROI with relatively uniform signal dynamics. In areas like the ventricular system, where the fluid dynamics differ throughout the region (Vikner et al., 2024), the technique’s assumption of coherent physiological oscillations becomes invalid. Fourth, vessel segmentation required PPG, leading to the exclusion of participants with poor PPG quality. However, hypersampling does not rely on PPG and can be applied to GM and WM in studies without. Lastly, with the 2 mm and 2.5 mm isotropic fMRI resolutions, vessel segmentations may contain small amounts of CSF due to partial volume effects. We confirmed that CSF contributes minimally to vessel fMRI signals, as it lacks the steady-state pattern seen in pure CSF voxels during the first few TRs (results not shown).

### 4.6. Conclusion

Our region-specific hypersampling technique (HyPER) overcomes the temporal limitations of conventional fMRI, enabling the recovery of spatially distinct physiological oscillations that were previously inaccessible. We demonstrated the feasibility, reliability, and broad applicability of this approach by applying it to the HCP-A fMRI datasets, where we identified age-related changes in physiological pulsations across brain regions. This method can be retrospectively applied to existing fMRI datasets, offering new opportunities to uncover novel insights into brain physiology beyond traditional functional connectivity. By revealing age-related physiological changes, our findings underscore the potential of this technique to advance our understanding of brain physiology and disease-related alterations.

## Supplementary Material

Supplemental Material

Supplementary material associated with this article can be found, in the online version, at doi:10.1016/j.neuroimage.2025.121502.

## Data Availability

The data came from the HCP-A 2.0 Release, DOI: 10.15154/1520,707. The study data is available for download through the NIMH Data Archive (https://nda.nih.gov/), and the code will be made available upon reasonable request.

## Data availability

The data analyzed was through a publically available dataset – HCP-Aging, but an application is required to acquire access to the datasets. Code will be shared upon request.
