# Supplementary material for: HyPER: Region-specific hypersampling of fMRI to resolve low-frequency, respiratory, and cardiac pulsations, revealing age-related differences
Source: Neuroimage. Author manuscript; Available in PMC 2026 May 10. (PMC13157684; doi:10.1016/j.neuroimage.2025.121502)
Supplement: Supplemental Material [file NIHMS2161162-supplement-Supplemental_Material.docx]

**Supplemental Material:**

**Results:**

- - 1. **Strong agreement in physiological power between hypersampled and fast fMRI.**

The ROI-specific comparisons between the power of the hypersampled fMRI and the original fast fMRI were consistent with the ROI-grouped comparison (Supplemental Figure 1 compared with Manuscript Figure 3b).


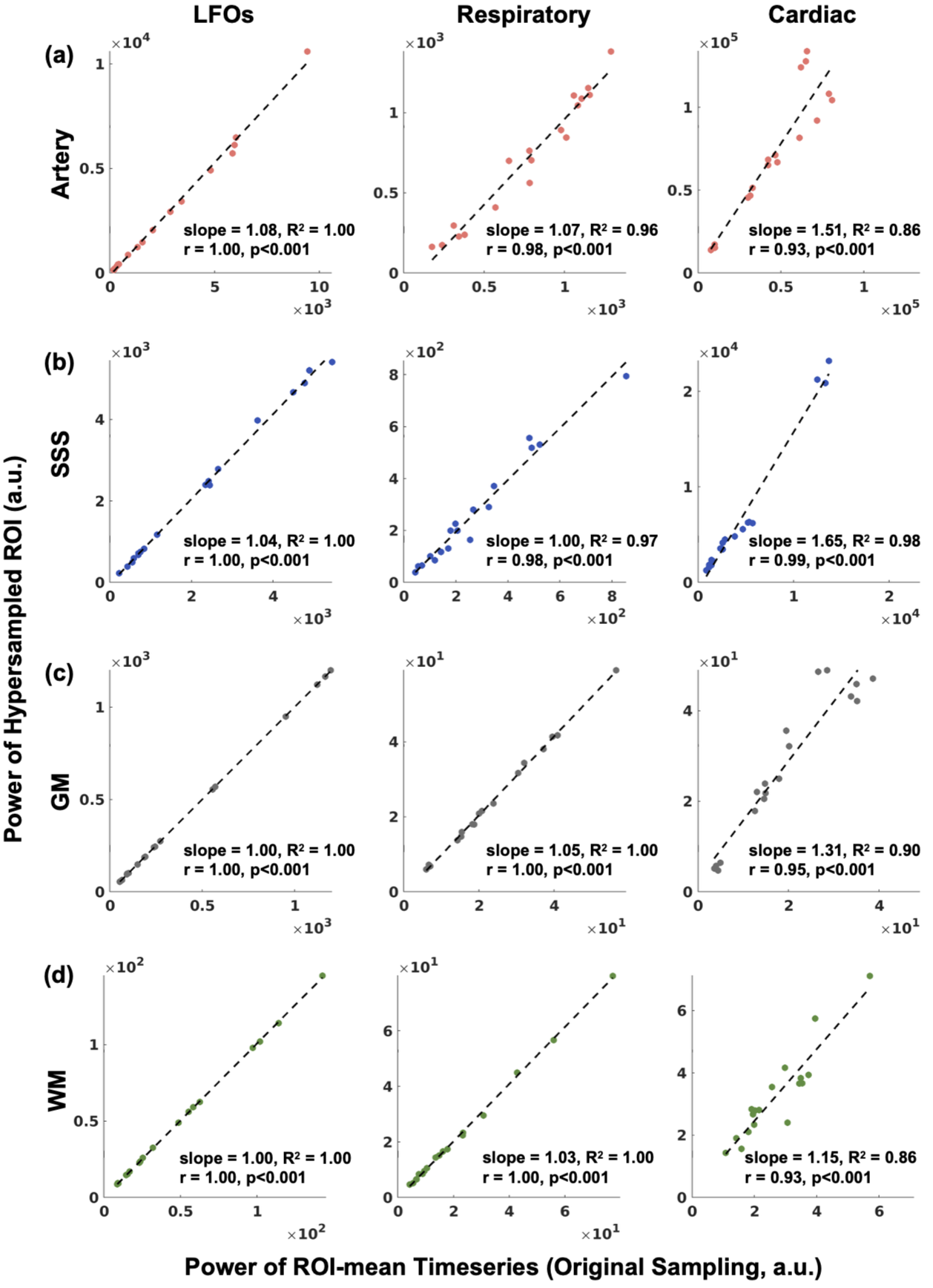


***Supplemental Figure 1:*** *A strong agreement in absolute power was observed between original and hypersampled data across all physiological frequency bands and regions of interest (*$r\geq0.93$ *for all comparisons, 19 total fMRI scans from 5 participants).* ***Left to right:*** *LFOs, Respiration, Cardiac,* ***Top to bottom: (a)*** *Artery,* ***(b)*** *Superior Sagittal Sinus (SSS),* ***(c)*** *Gray Matter (GM), and* ***(d)*** *White Matter (WM).*

**3.3.2 Cardiac power was greater in hypersampled fMRI compared to the ROI-mean approach because hypersampling accounts for slice timing.**

The cardiac power was consistently higher in the hypersampled fMRI compared with the ROI-mean timeseries (slope > 1, Supplemental Figure 1). The higher cardiac power observed in hypersampling compared with the original fast fMRI time series arises from its ability to better recover the signal oscillations by accounting for slice timing. In contrast, the ROI-mean operation ignores slice timing differences, leading to temporal averaging across slices. This averaging causes greater destructive interference for rapidly varying signals, such as cardiac pulsation, than for slower signals such as LFOs and respiratory oscillations. For example, in fMRI acquisitions, neighboring slices capture different phases of the cardiac cycle, so averaging across slices combines multiple cardiac phases that destructively interfere. Consequently, cardiac power is more underestimated in the ROI-mean signal.

In the following analysis, we show that the ROI-mean operation, by ignoring slice timing, is effectively equivalent to applying a moving average to the ground-truth signal, which reduces power at rapidly varying frequencies such as cardiac pulsations. Using hypersampled data as the ground truth, we applied a moving average to mimic the ROI-mean operation and compare the resulting physiological powers.

**Methods:**

Specifically, we compared the physiological power of the hypersampled fMRI with an approximation of the original fMRI signal (TR = 0.366 s). First, we calculated the physiological bandpower of the, referred to as the “Power of Hypersampled ROI”. Next, to approximate the original fMRI signal, we took the hypersampled timeseries, applied a moving average across the number of hypersampled points within one TR (NSlices/MB = 40/8 = 5), and downsampled to the original TR. This post-processing yields a timeseries that mimics the acquisition at the original sampling rate. The bandpower of the approximated signal was calculated and referred to as the “Power of Approximated Original-Sampled ROI”. Finally, we directly compared the power of the hypersampled ROI with the power of the approximated original-sampled ROI.

**Results:**

Across all ROIs, the physiological power measures from the hypersampled fMRI data were very strongly correlated with the power from the approximated original fast fMRI signal (Supplemental Figure 2, $r\geq0.99$, p<0.001). The LFO and respiratory power had regression slopes approaching 1 (slope=1.00 and 0.99, respectively), and the cardiac power slope was approximately 1.5. These slopes closely track with the slopes of the power of the hypersampled ROI vs. the power of ROI-mean timeseries (original fast fMRI, Manuscript Figure 3b). In both analyses, the LFO and respiratory power regression slopes approached 1, indicating proportional agreement between the powers. This suggests that lower frequency oscillations, such as LFOs and respiration, have long enough periods that the slice-to-slice phase difference is negligible when taking the mean using original slice timing. However, in both analyses, the cardiac power regression slopes approached 1.5, supporting that the higher cardiac power observed in the hypersampled fMRI is likely due to preserving the cardiac phase differences between slices.


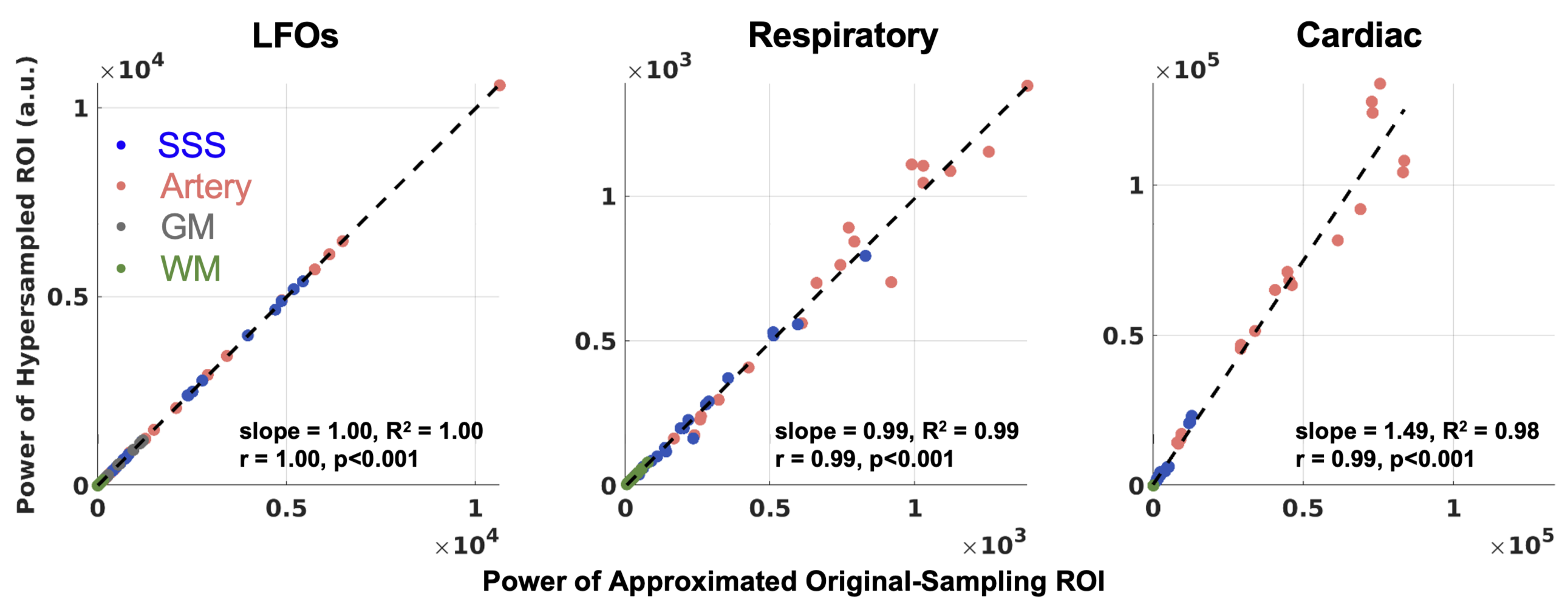


***Supplemental Figure 2:*** *Physiological power measures from the hypersampled fMRI data and the approximated original fast fMRI signal very strongly correlated (*$r\geq0.99$*) in all physiological frequencies and regions of interest.*

- 1. **Impact of pulse propagation on hypersampling in white matter.**

In white matter (WM), the hypersampled ROI power and the mean voxel-wise power were moderately correlated in the LFOs (r=0.63, p<0.01) and strongly correlated in respiratory (r=0.94,p<0.001) and cardiac power (r=0.85, p<0.001), see Supplemental Figure 3.


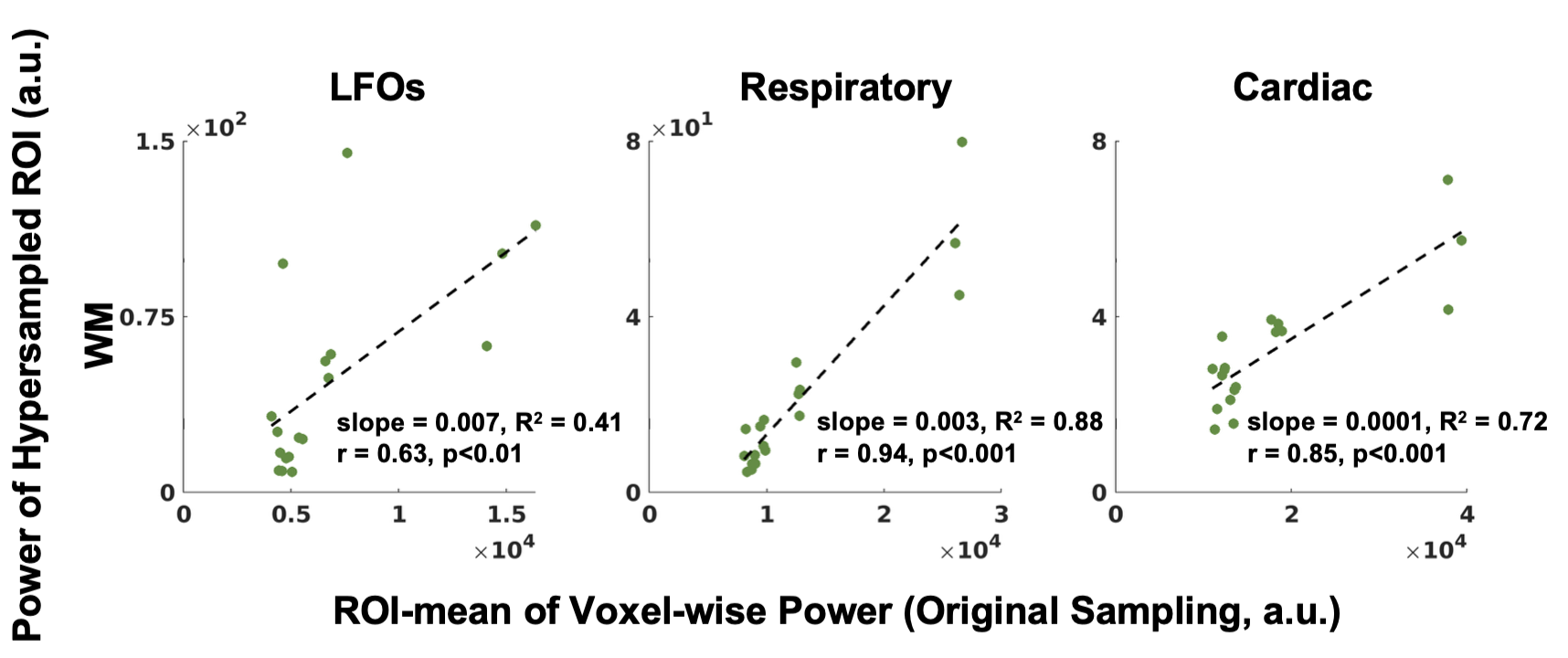


***Supplemental Figure 3:*** *Impact of physiological propagation in white matter (WM). The hypersampled ROI bandpower (Hypersampled ROI power) with voxel-wise physiological bandpower from the locally acquired fast fMRI (ROI-mean Voxel-wise Power) were moderately correlated in LFOs (left), and strongly correlated in respiratory (middle) and cardiac (right) power.*

- 1. **Age-related differences in hypersampled power spectra in HCP-A**

The younger (age = 49.7±8.2 years, N = 388, 41% men) and older (age = 75.7 ± 6.7 years, N = 221, 46.1% men) groups’ average power spectrum for white matter (WM) is summarized in Supplemental Figure 4a. The associations between age and physiological bandpower in the WM are summarized in Supplemental Figure 4b.


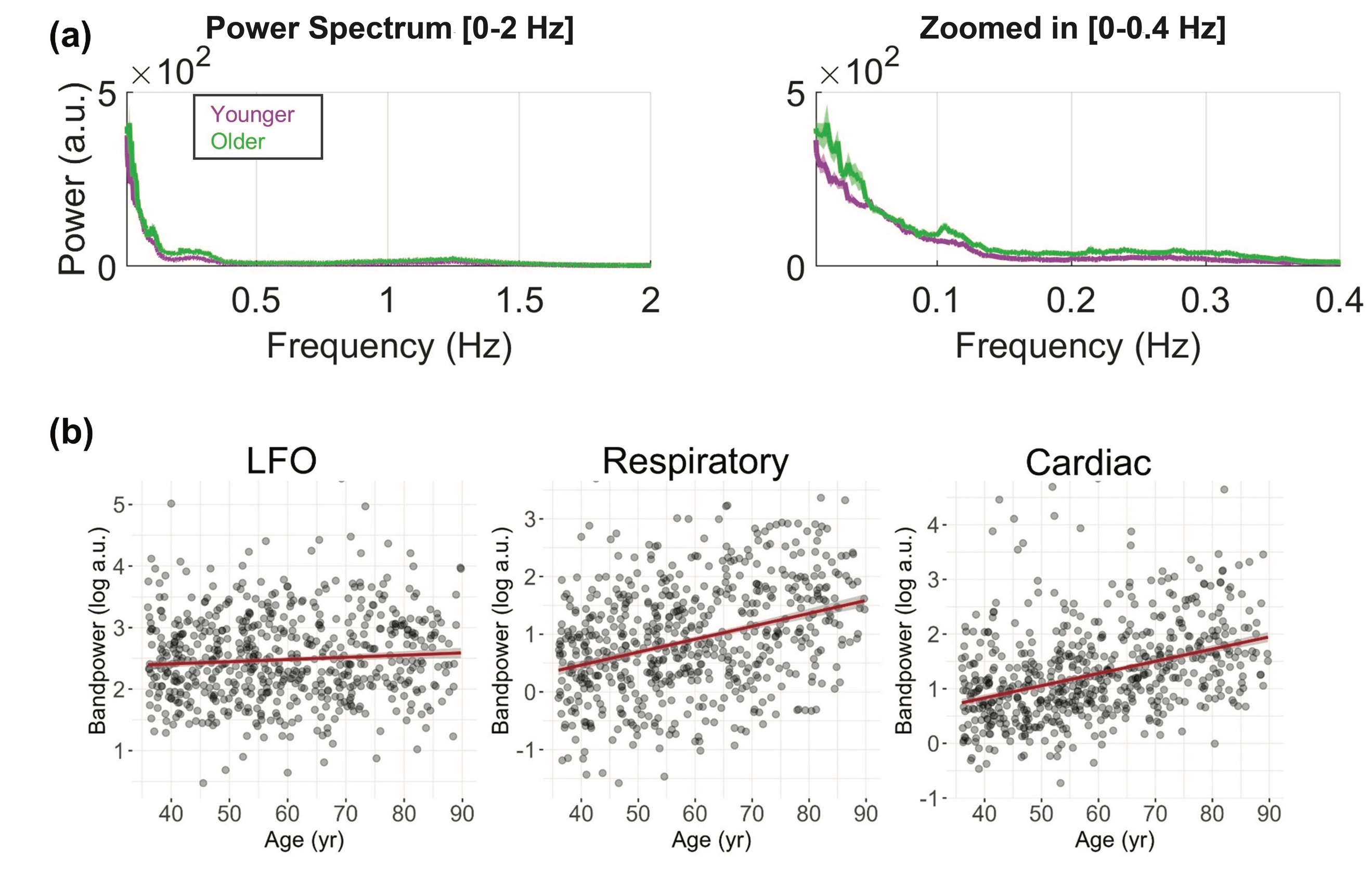


***Supplemental Figure 4:*** *Summary of physiological bandpower in white matter (WM) across age.* ***(a)*** *Comparison of physiological power spectra between younger (49.7±8.2 years) and older (75.7±6.7 years) participants in the WM. The mean (solid line) and standard error (shaded region) are displayed.* ***(b)*** *Age-related associations with white matter (WM) bandpower in low-frequency oscillations (LFOs), respiratory, and cardiac frequencies.*

All linear-mixed effects model statistics for age and biological sex as predictors are provided in Supplemental Table 1.

***Supplemental Table 1:*** *The model summary statistics for every linear mixed effects model, by region and physiological bandpower.*

|  | | Age | | Biological Sex | |
| --- | --- | --- | --- | --- | --- |
| ROI | Physiology Band | t-statistic | FDR corrected  p-value | t-statistic | FDR corrected  p-value |
| Arteries | LFO | 2.60 | 0.0113 | 4.18 | 0.0000 |
|  | Respiratory | 8.36 | 0.0000 | 0.51 | 0.6115 |
|  | Cardiac | 18.43 | 0.0000 | -5.22 | 0.0000 |
| SSS | LFO | -3.64 | 0.0004 | 7.72 | 0.0000 |
|  | Respiratory | 2.63 | 0.0105 | -0.70 | 0.4852 |
|  | Cardiac | 10.18 | 0.0000 | -4.63 | 0.0000 |
| GM | LFO | -2.01 | 0.0535 | 5.84 | 0.0000 |
|  | Respiratory | 8.11 | 0.0000 | 3.58 | 0.0006 |
|  | Cardiac | 10.60 | 0.0000 | -0.26 | 0.7965 |
| WM | LFO | 1.76 | 0.1172 | 5.05 | 0.0000 |
|  | Respiratory | 9.06 | 0.0000 | 1.12 | 0.2957 |
|  | Cardiac | 10.33 | 0.0000 | 1.05 | 0.2957 |

The comparison of region-specific physiological bandpower between biological sex is summarized in Supplemental Figure 5.


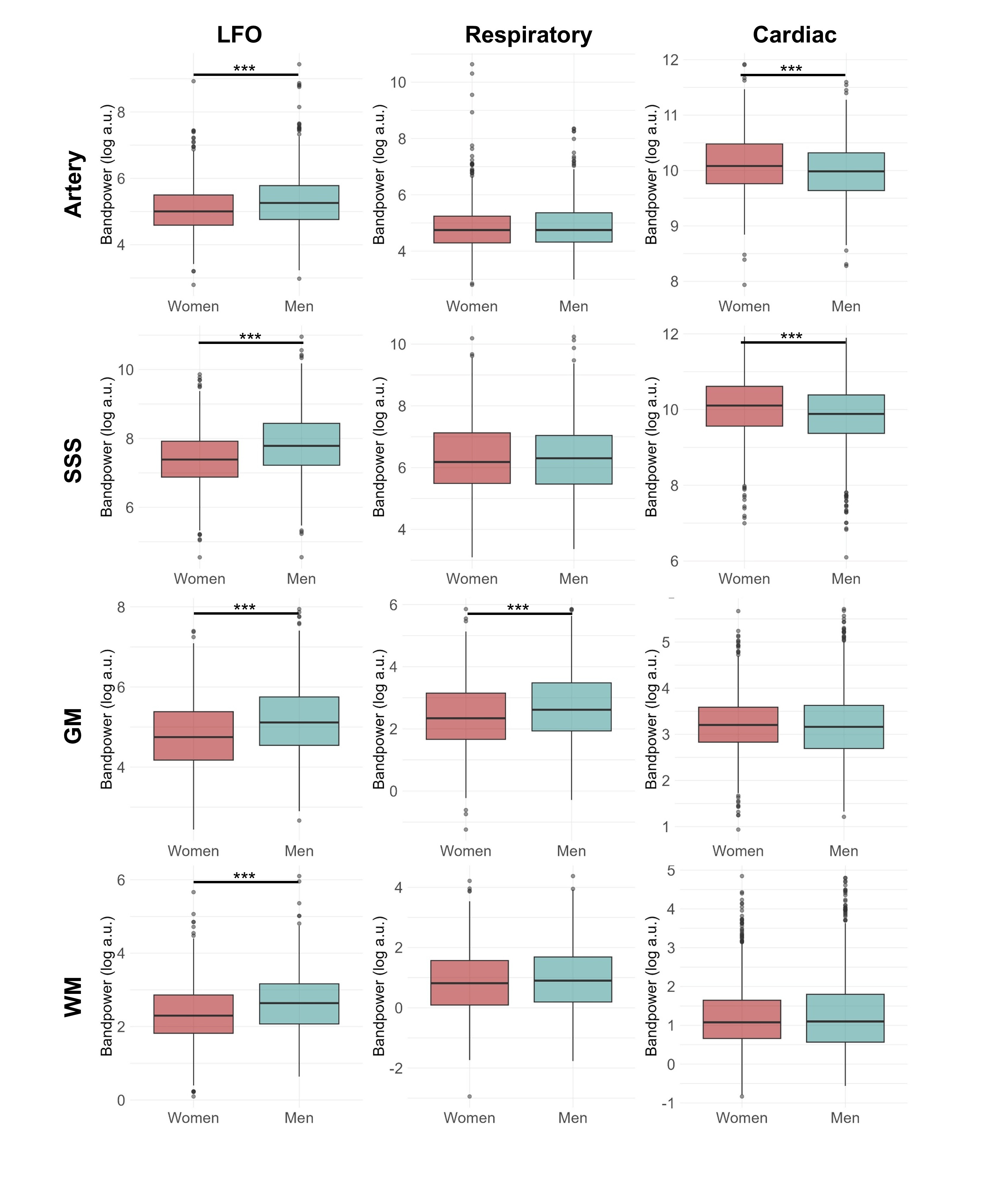


***Supplemental Figure 5:*** *Summary of physiological bandpower across arteries (first row), the superior sagittal sinus (SSS, second row), gray matter (GM, third row), and white matter (WM, fourth row), categorized by biological sex.*
